# “Heartbreaking, Hardest Part of the Job”: A Qualitative Descriptive Study of Acute Care Nurses’ Work with Patients with Dementia Who Self-Neglect Their Hygiene

**DOI:** 10.3390/healthcare13131562

**Published:** 2025-06-30

**Authors:** Patricia Morris, Rose McCloskey, Janet Durkee-Lloyd, Karla O’Regan

**Affiliations:** 1 Faculty of Nursing, University of New Brunswick, Fredericton, NB E3B 5A3, Canada; p.morris@unb.ca; 2 Department of Nursing & Health Sciences, University of New Brunswick, Saint John, NB E2L 4L5, Canada; 3 Department of Gerontology, St. Thomas University, Fredericton, NB E3B 9V7, Canada; jdurkee@stu.ca; 4 Department of Criminology & Criminal Justice, St. Thomas University, Fredericton, NB E3B 9V7, Canada; oregan@stu.ca

**Keywords:** acute care, dementia, qualitative research resistance to care, self-care

## Abstract

When nurses encounter people in institutional settings who are living with dementia and self-neglecting their hygiene, they are challenged to provide care that respects autonomy while upholding the ethical principles of beneficence and non-maleficence. This study aimed to understand how nurses respond when confronted with patients who decline assistance with personal hygiene and then became physically aggressive. **Methods**: A qualitative descriptive study employing think-aloud interviewing to explore nurses’ clinical reasoning about how they would proceed with the care of a patient living with dementia who self-neglects their hygiene. **Results**: Nurses describe many creative ways that they would work with patients to accomplish personal hygiene care in an ideal world. Participants also share the many barriers they experience to providing desired care and instances where they would force care with people who self-neglect their hygiene. Thematic analysis revealed six key themes: non-preferred approaches to care; preferred approaches to care; barriers to actualizing preferred approaches; responding to continued resistance to care; justified use of force; and efforts to minimize harm. **Conclusions**: This study highlights that ethical nursing practice in dementia care is not simply a matter of following through with best practices. It is an ongoing negotiation, carried out in environments that are often misaligned with nurses’ values.

## 1. Introduction

In the context of dementia, nurses have long been challenged to provide care to patients who decline assistance with personal hygiene care but are prone to “self-neglect” [1]. Self-neglect is characterized by a new or worsening inability to perform essential activities of daily living related to food, hygiene, living environment, and personal safety that were performed before the onset of dementia [2]. It can involve improper toileting, reduced or ineffective bathing, hoarding or improper home maintenance, medication mismanagement, financial mismanagement, and more. Self-neglect can pose a serious threat to older adults’ health and psychosocial well-being [3,4]. Self-neglect places older adults with dementia at risk for early mortality, and it can have deleterious effects on overall health and well-being if left unaddressed [5].

Nurses caring for individuals living with dementia who self-neglect in hospitals are frequently required to provide hygiene care, including bathing and toileting, even when the person resists or refuses assistance [6,7]. These situations present significant ethical and clinical challenges. Nurses must navigate the tension between respecting the autonomy and dignity of the individual and addressing immediate concerns related to health, safety, and institutional expectations [8]. Decisions to withhold, delay, or proceed with hygiene care are not taken lightly [7]. Nurses may attempt to change their approach in order to gently persuade the individual to receive care [9,10], but there may also be cases in which the refusal is ultimately respected—even when the decision to do so may result in risk to the individual [11]. While clinical resources often conceptualize these decisions as rational processes grounded in weighing risks and assessing the patient’s capacity to make risky decisions [12], there is limited empirical research examining how nurses engage in this reasoning in practice. Specifically, little is known about the cognitive, emotional, and contextual factors that influence nurses’ decision-making in moments of care refusal and self-neglect.

This qualitative descriptive study addresses this gap by engaging nurses in think-aloud interviews [13] to elicit rich, first-person accounts of their clinical reasoning. By foregrounding nurses’ own voices, the study offers a nuanced understanding of how decision-making unfolds in real time—revealing processes shaped not only by clinical judgment, but also by ethical commitments, institutional constraints, and the relational dynamics inherent to dementia care. In doing so, the study sheds light on how nurses negotiate competing obligations, interpret ethical principles in context, and navigate the complex structural and interpersonal landscapes that define their everyday practice.

## 2. Materials and Methods

### 2.1. Research Questions

This study was guided by the following research question: When presented with a scenario involving an older adult with dementia who self-neglects and declines assistance with hygiene care, what possibilities for action do nurses identify, and what reasoning processes do they draw upon to support their decision-making? To further explore this phenomenon, two sub-questions were developed: (1) How do nurses describe their preferred approaches to providing hygiene care in this situation? and (2) What approaches do nurses report avoiding or disavowing, and why? These questions were designed to elicit nuanced descriptions of clinical reasoning, values, and ethical considerations in the context of hygiene care for persons with dementia who refuse assistance.

### 2.2. Study Design

This study was a qualitative descriptive design. The COREQ checklist [14] was consulted for comprehensive reporting, and it is included as a Supplementary Document (see Supplementary Material Table S1). In this study, think-aloud interviewing was employed as a data collection method to explore nurses’ decision-making processes in the context of providing hygiene care to patients with dementia who self-neglect and decline assistance. Originally developed in psychology, the think-aloud method enables researchers to examine cognitive processes by asking participants to verbalize their thoughts as they solve a problem [15]. These interviews can be conducted in real-time, as participants complete a task, or they can proceed after exposure to a standardized case scenario [16,17]. Although case scenario approaches may limit access to real-time cognitive data, they allow for standardization that provides valuable insights into patterns of reasoning and can provide rich and evocative prompts that stimulate imagination without the pressure of immediate decision-making [18]. In this study, participants watched a short video which simulated a nursing hand-off report about a patient who declined assistance with personal hygiene care and became physically aggressive when pushed to continue with care provision (see Supplementary Material Table S2 for the video script). Think-aloud interviews align with the qualitative descriptive design by capturing a clear, detailed account of the participants’ experiences without imposing theoretical frameworks or predefined analyses. By encouraging nurses to articulate their reasoning, the technique provided an understanding of how they evaluate and respond to challenges in dementia care, ensuring that the data remained grounded in the participants’ lived experiences.

### 2.3. Participant Selection and Interviews

Thirteen registered nurses (RNs) and five licensed/registered practical nurses (LPN/RPNs) volunteered to participate in this study. There was no attrition and no one declined to participate after the informed consent process was carried out. Demographics are described in Table 1.

Participants were recruited using purposive sampling to ensure a range of experiences among RNs working in acute care hospitals. Participants were informed that the research team was conducting interviews about the clinical reasoning involved in working with patients with dementia in hospital settings who decline assistance with hygiene care. Inclusion criteria included (a) being a registered nurse or licensed practical nurse; (b) working in an acute care hospital in the last five years; (c) having experience providing care to older adults with dementia in hospitals; and (d) encountering at least one instance of care refusal involving hygiene. Recruitment was carried out through a promotional flyer posted on popular social media sites (Facebook and Instagram), as well as a snowball sampling technique [19]. Interviews were conducted virtually via Zoom, allowing for flexibility in participant geography. Participants joined from private locations of their choosing, ensuring confidentiality and comfort during interviews. The interviewer conducted interviews from her office on campus with a blurred virtual background. No one else was present during the interviews. Interviews lasted between 45 and 75 min, were audio-recorded with consent, and transcribed verbatim.

Interviews were conducted in the summer of 2023 using a semi-structured guide with room for probing (see Supplementary Material Table S3). The researchers had no prior therapeutic or supervisory relationships with participants. All interviews were conducted by the lead author, a female RN and PhD-prepared nursing faculty member in Canada with over ten years’ experience in gerontological nursing. Her shared professional identity with the participants may have supported rapport; to address potential bias, she engaged in ongoing reflexivity throughout data collection and interpretation, attending to how her background and assumptions might shape the research process [20]. While her clinical and academic expertise in dementia care provided valuable contextual understanding, it also carried the risk of influencing how participants’ responses were interpreted. To mitigate this, she adhered closely to the semi-structured interview guide and used open-ended, non-leading questions, allowing participants to direct the conversation based on their own experiences. Field notes were recorded after each interview and memos were written at key stages throughout the analysis process to support transparency in analytic decisions. Regular peer debriefing with members of the research team (two of whom are non-nurses) helped ensure alternative interpretations were considered [21].

Recruitment and interviewing continued until thematic sufficiency was achieved, which was defined as the point at which no new conceptual insights were emerging from the data [22]. This was determined through an iterative coding process in which the research team reviewed transcripts concurrently with data collection and monitored the process for coding stability and repetition of themes. The final interviews yielded no novel codes or significant modifications to the existing thematic structure, indicating that thematic sufficiency had been reached.

### 2.4. Ethics

Ethics approval was obtained from the first author’s university’s Research Ethics Board (REB-2023-2017). Participants provided informed consent prior to commencing interviews and were assured of confidentiality, the voluntary nature of participation, and the right to withdraw at any time. Given the potentially emotionally complex nature of discussing ethically challenging care scenarios, participants were informed of possible emotional discomfort and provided with information on supportive resources should they experience distress. No participants reported distress during or after the interviews.

### 2.5. Data Analysis

Data analysis followed an iterative process consistent with qualitative descriptive methodology [23]. The first author led the analysis, which involved multiple readings of the transcripts, development of initial codes, and constant comparison across interviews. MAXQDA (Verbatim Inc., Berlin, Germany) was used to assist with data management and coding. Codes were then grouped into categories and refined into themes by two independent researchers (the first and second authors) [23] and represented hierarchically using coding trees. Trees were combined through discussion and consensus with the entire research team. Credibility was enhanced through member checking, where participants were invited to review summaries of their interview data and selected themes. While no participants chose to engage in this optional review, the opportunity to participate in this validation process was made available to all. No repeat interviews were performed. Dependability was supported by maintaining an audit trail of coding decisions, memos, and analytic drafts [24].

## 3. Results

### 3.1. Theme One: Non-Preferred Approaches to Care

Participants began each interview by offering their critiques of the case scenario nurses’ actions. They described the patient as someone responding quite typically for his age and dementia status and, given what they know about best practices in dementia care, they would have responded to the patient differently from the nurses in the case scenario. They identified 18 different critiques of the nursing care described in the case scenario (Table 2).

The most popular critique was that the nurses in the case scenario failed to assess the patient’s cognition (n = 12; 66.7%). The person’s cognition and ability to appreciate the danger of their self-neglect was paramount for participants. For example, Donald shared that the first thing he would do in this situation would be to do a “kind of cognitive-cognitive assessment”. He emphasized that only after he had completed his cognitive assessment would he move into careful planning about how to approach the patient. Evangeline also emphasized the importance of cognitive assessment and shared that she “would have started with you know, all the general orientation assessments. Um, you know, first of all, he’s not understanding where he is or why he’s there, then that’s going to be something to explore. If he doesn’t know who I am, or why I’m in the room, might be exploring that as well. You know, those types of things: orientation would be a big one and where he’s at with his plan for the day”.

For Evangeline, it is important to assess general orientation and to explore his perceptions of what is going on and what he plans for himself for the day. Failure to do this means that she would not know “how receptive he is to me and what I’m saying and if he’s understanding kind of the situation”. Ella, Nancy, and Stella also emphasized assessing his understanding of the situation and noted that it is crucial to assess how advanced the patient’s dementia is before deciding on a course of action. Participants understood assessing capacity as critical to deciding how to proceed, and whether care might reasonably be foregone for the time being.

Related to that cognitive status, participants emphasized that a critical error in the case scenario staff’s approach was that they tried to do the patient’s care in the evening (n = 11; 61.1%). Participants found the provision of hygiene care in the evening problematic because of behavioral patterns they would expect from someone living with dementia. For example, Nicole described the patient as “having a shift, just a natural shift with the sundowning” which made providing care challenging. Stella also described the evening as a time when things “shift” for patients living with dementia: “There’s some people with dementia, where it’s kind of like getting me thinking about, like, the whole sundowning thing. Like, during the day they’re very clear, and you might not really realize that they have dementia, and then at night, like, things sort of shift”.

Participants perceived care to be much more challenging during those shifts because the person has transitioned from a state of rationality to a state where “they don’t have the best judgement for themselves” (Stella). As such, they may become more resistant to care in the evenings. Margaret noted her surprise that the staff in the case scenario did not seem to recognize these patterns as quite typical of people living with dementia. She shared, “I don’t understand why they don’t expect that to happen, I guess. The mood, the mood changes and things should be expected”. For Margaret, staff who understand the patterns of cognitive impairment ought to understand that patients who are confused or live with dementia behave in particular ways as night approaches and especially as personal care is offered to them. She went on to say, “people with dementia are never good in the evening or overnight, so that wouldn’t be the best time to approach them for sure”. According to the participants, people with dementia often behave in particular and predictable ways that the staff in the case scenario failed to appreciate.

Participants were not all in agreement about the best way to approach the case scenario patient. For example, some participants wished that the nurses in the case scenario had not jumped to chemical restraints so quickly, and others wished that the nurses had pursued this option earlier. One nurse felt that the nurses in the case scenario went back and tried to persuade the person too many times, while another felt the nurses underestimated the urgency of the situation and should have kept returning until care was complete. When I asked participants what they might have done instead, the answers were similarly divergent.

### 3.2. Theme Two: Preferred Approaches to Care

Participants described 20 distinct ways that they would approach the patient and try to creatively work around his resistance to get him washed (see Table 3). Most participants described using multiple strategies at once and changing strategies to suit the individual patient’s needs. Finding the right strategy for the individual patient—“what works best for them” (Angela)—was a priority.

The most common approach that participants described was to decrease the patient’s overall agitation by administering a PRN (pro re nata, meaning “as needed”) medication or to wait until scheduled behavioral medications had time to work. Sophie shared that often when patients exhibit RTC, “we kind of wait for their either their PRN medication or their evening meds to work before we would change them and put them to bed”. Alexa similarly noted that the patient’s care “should have almost been dealt with early on that like, it’s like, ‘hey, maybe we need a PRN’, you know? Some kind of chemical sedation maybe to help with him”. Karen described PRN medications as a way “to kind of smooth their emotions, kind of help them—help them chemically to kind of get into a different mindset that so that, that they are more, um, comfortable with the situation or are able to become more comfortable situation”.

Participants were clear that this medication was to help and soothe the patient, though, and not simply a medication that they administered to complete care.

Another common approach that participants proposed was to give the patient time to get to know them. Participants felt that, as the day wore on, the patient would become more comfortable with the staff. Angela shared that she would focus on “getting him comfortable with me as a person first” before she attempted to provide personal care. Sophie emphasized not “jumping the gun” and offering care on their first meeting, and Karen shared the importance of going in softly, not “full guns blasting”. Alexa shared the importance of waiting instead of “rampaging in”. Participants also recognized that there might be some cases where, even if they do wait, the person may never be comfortable enough with them to allow assistance with personal care. Other strategies to persuade the patient to accept assistance with care were asking for help from family caregivers who had been managing the patient’s care in the community (n = 8; 44.4%), explaining the risks of declining care to the patient (n = 8; 44.4%), attending to setting events (e.g., hunger, fatigue, pain) (n = 5; 27.8%), dividing the bath up into many parts (n = 4; 22.2%), and doing the person’s care in bed so that they are less mobile and cannot flee (n = 1; 5.6%). Four participants described drawing on the physician’s authority to accomplish care. While participants were clear that the doctor/nurse hierarchy was problematic, they were also willing to use the power differential to their advantage as needed. For example, Joy described using a doctor’s authority along with therapeutic irreverence when assisting patients with personal care. She explained: “Some people take it okay and some people get mad, but I’m like, ‘your butt smells. We have to do something here. Doctor can’t smell that, he’ll be upset.’” This approach, while direct and seeming to verge on unprofessional language, reflects a pragmatic, relationship-based strategy for overcoming patient resistance to care that also leverages the authority of the physician.

### 3.3. Theme Three: Barriers to Actualizing Preferred Approaches

Whatever approach they preferred to take to the patient’s care, participants were clear that they wanted to care for this patient well and with attention to personal preferences. Overwhelmingly, though, participants shared that they are unable to provide this kind of care because they just do not have time. For example, Nicole stated that providing care the way that patients with dementia need is impossible in acute care. She said, “taking time to spend a lot of energy and in a sensitive manner in the way that they are going to respond to is not something that I have time for”. Stella noted that, “realistically, like that’s—it’s really hard to provide that kind of care in a time crunch scenario, like most acute care situations are”. What nurses *do* need to make time for is what participants called their “checklist” (Anna, Donald), their “priority list” (Nicole), their “to-do list” (Astrid, Nancy, Evangeline), or “the list of things that are just like, that keep creeping up” (Alexa). The content of the to-do lists varied slightly from participant to participant, but usually included initial assessments (e.g., vitals, wound assessments, skin assessments), medication provision, and addressing acute health challenges.

Participants shared that washing patients was constantly pushed lower on their priority lists as their lists became crowded with administrative tasks and sicker and sicker patients. Participants were even able to list the parts of the patient’s body they would prioritize in a bath to do just what was necessary to save on time. Alexa noted that “the essentials”, for her, are “the armpits, teeth, bums”. Ella and Sophie both emphasized the importance of at least completing “good peri-care”. For non-nursing readers, the term peri-care is nurses’ shorthand for perineal care, which describes a specific procedure for cleansing a person’s perineum (the area around the genitals and anus). Anna described the bare minimum of bathing as giving patients “a wipe and a swipe”. According to her, the key areas that should be cleaned are “bums and gums”.

### 3.4. Theme Four: Responding to Continued Resistance to Care

When asked what they might do if the patient continued to decline their care, despite their best efforts to encourage them, participants’ responses were varied and often uncertain. Astrid jokingly responded to my question by saying, “You’re not making this easy. Oh, oh, what am I gonna do? I don’t know”. Jesus exclaimed, “Oh my god. Declining care is the hardest thing”. Anna shared that for her, “That’s a big deal. That’s really tough”. Most participants interpreted this question to be about whether they thought that the use of force was appropriate in this context, and most were adamant that force was not a frontline approach used by good nurses.

Participants described force in vivid terms: “rampaging in” (Alexa), “holding people down” (Margaret), “tying them in” (Joy), “strapping” them to the bed (Nancy), “ganging up on them” (Karen) and a whole manner of options that they felt should be left as a “last resort” (Anna). Participants often presented force as the antithesis of good nursing. For Nancy, force makes things worse. She shared how important it is:

“You know, just being engaging, and encouraging, and helpful, but not forceful. Because I don’t think being forceful helps. I think that, generally speaking, that just sort of makes things worse, it gets people agitated, more than anything, because, I mean, I’m sure if someone was in that situation, they don’t want to be in it either. So just, yeah, being supportive. ”

Several other participants (Nicole, Anna, Margaret, Evangeline, Donald, Alexa) postulated about how the use of force might impact their therapeutic rapport with patients. These participants explained that using force often increases agitation and will almost certainly erode the caregiving relationship. Anna described the devastating impacts of forcing personal care:

Your entire therapeutic trusting relationship is completely demolished. You now take pills into this person—are they going to trust that they’re the right pills? Are they going to trust that you’re not poisoning them? Are they going to trust that you have their best interests at heart? Absolutely not. “If she’ll force me”—pardon my language, but “if she forced me to open my legs, what else is she gonna force me to do?”

The raw and unsettling language in Anna’s account—particularly the phrase “if she forced me to open my legs, what else is she gonna force me to do?”—evokes a profound sense of violation and mistrust. The connotations of sexual violence and coercion underscore how deeply vulnerable nurses feel they render people through the provision of personal care. Her rhetorical questions highlight the potential for the caregiver to become intelligible as a threat, rather than a trusted ally and helper. While not all used such evocative language, participants all decried the use of force unless absolutely necessary. The one instance in which all participants felt it would be necessary is fecal incontinence.

### 3.5. Theme Five: Justified Use of Force

Fecal incontinence was the singular circumstance in which participants would intervene with force. For example, Sophie shared that, if someone is not incontinent, “I don’t get too upset”, but in the case of fecal incontinence, that’s when “you need the team to come in and do it”. For Margaret, too, fecal incontinence was the point at which the creative practices outlined above fell short: “It depends if they’re incontinent or not. If they’re going into the bathroom, it’s not a big deal. If they’re incontinent it is. That changes everything, right?” Nancy similarly shared that force is inappropriate in the context of personal care, unless someone had been incontinent: “So you wouldn’t, you wouldn’t force care in that context. Not unless they were incontinent and dirty”. According to Beatrice, “If they’re incontinent, I mean you can only allow that to go on for so long, right?”

When asked how long it might be appropriate to wait if a patient continued to decline assistance but had been incontinent, participants’ answers varied. Sophie said, “oh, I wouldn’t think very long”. Stella answered simply, “I don’t know”. Others had a more exact answer. Astrid stated, “I don’t think it’s acceptable at all. Once you know, once you find that, you deal with it”. Bethany proposed that it might be acceptable for “maybe an hour at most”. Margaret similarly shared, “not long. My tolerance? Less than an hour”. Beatrice distinguished between urinary incontinence and fecal incontinence explicitly in her discussion of timing. She reasoned that incontinence products are designed to hold 12 hours’ worth of urine and absorb and wick that urine away from the skin, and so patients experiencing urinary incontinence need to be changed at least twice a day. In the case of fecal incontinence, though, she states, “my threshold for allowing that to continue would be much shorter. So I’m looking at maybe an hour, on the outside”.

Thirteen participants spoke directly about the various risks of unmanaged fecal incontinence (Table 4). Skin breakdown was the most common risk noted (n = 13/100%). This was followed by concerns about the risk of infection (n = 10; 76.9%), with some participants noting infection in general was a risk while others mentioned urinary tract infections explicitly. While participants’ acknowledged skin breakdown and infection as common side effects of unmanaged incontinence), the threat of unnamed risks also worried participants’ discussions of incontinence. While participants often began by listing specific risks, they sometimes concluded with non-specific, unnamed implications of incontinence. They stated that incontinence can lead to “skin breakdown and infection and all that stuff” (Sophie), or they argued that, “if he’s, like, continuously soiled in school, that’s gonna give skin breakdown and like that kind of problems” (Astrid). After Margaret named bladder infections and skin breakdown as risks, she went on to state that incontinence is “just not good for the health of anything in that region, I guess”. Angela, similarly, notes several specific risks and then states that she would generally be “worried that that person’s health was—there was going to be a potential implication towards this person’s health” if incontinence were left to persist. Joy, too, shared that she worries about nursing home residents who are admitted to hospital because “I’ve seen what can happen if they don’t turn them. They don’t wash them. They don’t—they don’t wash them. They just don’t”. Allusions to health in general, references to the sights that they’ve seen, and phrases like “all that stuff” allude to potentially bigger problems without actually naming them.

### 3.6. Theme Six: Minimizing the Harm

In order to minimize the harm they felt they were doing by providing unwanted care by forceful means, some participants emphasized that they move as quickly as possible to complete the person’s hygiene care (Angela, Nicole, Astrid, Alexa, Margaret, Sophie, Ella, Jesus). The rationales they provided for moving quickly are outlined in Table 5. The most common reasons for working quickly were simply to get the experience over with as soon as possible (n = 5; 62.5%), and because the acute care environment necessitates moving quickly (n = 3; 37.5%). Interestingly, three participants emphasized that by moving quickly they were exposing the patient to less trauma than they would if they moved slowly (n = 3; 37.5%). One participant disagreed with this assessment wholeheartedly, though. Anna was quick to point out the flawed logic of completing care quickly in order to protect the patient:

Um, so some nurses, say you’re a charge nurse or whatever, some instructors, charge nurses, care providers would just say, “Well, you gotta do it. So just go in there. And I’ll hold his legs open for you. And we’ll do it as quickly as possible. So it’s, we don’t—we’re not violating them for 30 min. It’s only 15”. So that makes us feel better.

Despite Anna’s assessment that moving quickly makes nurses feel better, many participants shared how deeply it troubles them to provide unwanted care, even when they understand their actions as necessary. Some participants described themselves as feeling guilty or feeling generally “bad” (or a close synonym) about what they had to do to care for their patients. Angela shared that, “you feel terrible doing it”. Alexa shared that, “I feel bad that we had to go that far. I feel kind of guilty having to put them in it [restraints]”. Ella, too, said, “you feel bad, because you’re hurting them”. For most participants, the way that patients interpreted their forced care was the piece that impacted them most deeply. For Jesus, for example, declining care is “the hardest thing” because he finds that the patients “feel that you are abusing them, you are touching them, right. Um, but they don’t know what you’re doing. So it’s really hard”. Margaret, too, shared that “you feel like you’re violating their human rights or something, like, it’s just, just awful. Nobody leaves that situation feeling good”. Bethany also shared that it feels like “you’re just going against their human rights”. For Ella, deciding whether or not to force care in the context of hygiene self-neglect is “heartbreaking, hardest part of the job”.

## 4. Discussion

When older adults with dementia self-neglect in hospital and then decline assistance with personal hygiene care, nurses are challenged to strike a balance between respecting the person’s autonomy (the person’s right to self-rule) and practicing according to the principles of nonmaleficence and beneficence (the professional responsibility to prevent harm and promote the patient’s greatest good) [25]. Nurses are impelled by professional ethics and institutional standards to take responsibility for the well-being of patients with diminished competence who are incapable of caring for themselves [12], and yet they are also impelled to uphold the person’s autonomy as much as possible [26,27,28,29]. As nurses weigh equally important ethical principles against one another in their care of older adults with dementia who self-neglect, it would be tempting to conclude that they must arrive at a kind of stalemate about how best to proceed. In practice, though, the participants in this study were clear that nurses cannot sit idly by and watch as a person’s health and well-being deteriorate. In some cases, this meant trialing alternative approaches to gently persuade the person to comply. Changing the caregivers’ approach is a popular topic of consideration in the nursing literature [10]. Many interventions have been trialed to decrease or prevent the emergence of resistance to care, including singing while care is rendered [30], showing people photos of positive stimuli during care [31], playing videos of family members encouraging their loved one to get washed [32], and aromatherapy [33,34]. Participants drew, whether consciously or not, on many of the foundational principles for high-quality care that is articulated in this body of evidence for support when they described their preferred approaches to care.

The findings from this study also shed light on the tension between nurses’ ethical aspirations and the practical realities of acute care nursing in Canada. The accounts provided by participants reveal that, despite their desire to attend to patients living with dementia in ways that support their well-being, they frequently encounter systemic constraints to enacting these approaches. Time pressure and task prioritization, in particular, compromise their ability to nurse the way that they think they ought to nurse. Participants’ reflections on the “bare minimum” of hygiene care—focusing on “bums and gums”—may appear pragmatic, but it also speaks to a troubling recalibration of care standards driven by institutional priorities and economics [35]. These findings echo longstanding concerns in the literature that the structure of acute care often disincentivizes or devalues relational work with people living with dementia, in favor of routinized care that saves time and money [36,37]. The findings from this study suggest that the prioritization of technical tasks over relational care is not only fragmenting day-to-day nursing practice, but also reshaping nurses’ cognitive processes and influencing their speculations about how they might proceed in ethical decision-making.

A central tension raised in this study concerns the limits of patient autonomy, particularly for people living with dementia; about what counts as necessary care and who gets to decide. While most participants rejected the use of force as incompatible with good nursing, all identified situations—namely, fecal incontinence—where intervention without consent became justifiable or even necessary. This conditional approach to autonomy reflects what theorists have called “relational autonomy” [38,39,40], where the nurse’s responsibility extends beyond honoring refusal to include assessing the patient’s capacity and anticipating the consequences of inaction. Yet, this responsibility is fraught: nurses are asked to act as both protectors and enforcers, sometimes within the same encounter, and often without institutional support [6].

The ways nurses sought to minimize harm when compelled to act against patient wishes—by working quickly, expressing guilt, or rationalizing their actions—highlights the “moral residue” that accumulates in such situations [41]. These strategies, while pragmatic, reflect a deeper discomfort in decision-making that clings to nurses even as decisions are made quickly and decisively. This discomfort, and the strategies nurses use to live with it, speak to the emotional toll and ethical burden of practicing under conditions that routinely force trade-offs between professional ideals and institutional demands. This has been a concern in care of older adults for more than 30 years [42]. The presence of moral residue—those lingering feelings of guilt, regret, or unease—suggests that these are not simply decisions made and forgotten, but rather ethically loaded moments that accumulate over time and may contribute to moral distress or erosion of moral agency [8,43,44,45,46].

### Recommendations

There is a role for nurses in advocating for more ethical care for people living with dementia. This includes voicing dissent when care marginalizes patients, and when it prioritizes institutional efficiency over human connection, dignity, or autonomy. Nurses must, for example, push back against the assumption that expedited hygiene care is always in a patient’s best interest, and they must question policies that treat refusals of care as behavioural problems rather than complex ethical events. While such critical engagement has long been encouraged in the literature [47], it remains uncommon in practice. This is often because nurses lack the education and institutional supports needed to challenge the status quo without fear of reprisal.

Institutional policies must move beyond rhetorical commitments to person-centered care by embedding ethical responsiveness into the structure of care delivery [48,49]. This includes establishing staffing ratios that allow time for relational care and ethical deliberation [50], integrating ethical consultation services into routine clinical practice [51], and formally recognizing refusals of care as complex ethical events rather than behavioral challenges to be managed. Hospitals and health authorities should implement and adapt ethical decision-making frameworks tailored for use with older adults [52]. This means ensuring that they are embedded in clinical protocols around caring for people living with dementia, and making them accessible during point-of-care decision-making. Such integration would provide nurses with structured support to navigate the complex balance between autonomy and beneficence in dementia care, reducing moral residue [47] and enhancing ethical practice.

The findings of this study also underscore the need for educational supports to enable ethical dementia care in acute care settings. There is a need for robust ethics-focused content in pre-licensure curricula [53]. This would ideally include specific education on care refusal [54], capacity [55], and communication approaches tailored to individuals living with dementia [56]. Research also suggests that integrating strategies to prevent moral distress early and consistently within pre-licensure nursing programs can strengthen nurses’ resilience on graduation—especially when supported by comprehensive instruction in nursing ethics [57,58]. Continuing professional development programs should also prioritize ethical decision-making in dementia care, to continue enhancing nurses’ capacity for this type of care [53,59,60].

## 5. Conclusions

The participants in this study demonstrated thoughtful, compassionate reasoning and a desire to protect the dignity of their patients. Their accounts also reveal that ethical nursing practice in dementia care is not simply a matter of following through with best practices. It is an ongoing negotiation, carried out in environments that are often misaligned with nurses’ values. As such, ethical decision-making in acute care dementia nursing is not only an individual cognitive process—it is profoundly systemic. Addressing the challenges raised in this study will require not only better training and ethical reflection tools, but also structural changes that support nurses in doing the kind of work they are committed to doing: care that is ethical, relational, and deeply human.

## Figures and Tables

**Table 1:** Participant demographics (*n* = 18).

**Professional designation**	
Licensed/registered practical nurse	5
Registered nurse	13
**Years of nursing experience**	
>10	5
<10	13
**Gender**	
Man	2
Woman	16
**Race**	
Latina/o/x	1
Black	1
White	16
**Employment status**	
Full-time	14
Part-time	3
Retired	1
**Area of specialization**	
Medical/surgical nursing	9
Intensive care	2
Emergency medicine	4
Neurology	2
**Geographic distribution**	
Urban	13
Rural	5

**Table 2:** Critiques of nurses in the simulation (*n* = 18).

Participant (*n* = 18)																			
Critique.	Angela	Nancy	Anna	Astrid	Joy	Sophie	Nicole	Alexa	Margaret	Ella	Bethany	Shaina	Evangeline	Beatrice	Nancy	Donald	Jesus	Stella	Total (n/%)
Failing to assess cognition and the root causes of behaviors	X	X	X	X	X	X				X			X	X	X	X		X	12 (66.7%)
Doing the care in the evening					X	X	X	X	X	X	X	X	X	X				X	11 (61.1%)
Ineffective communication with patient during care			X	X							X		X		X			X	6 (33.3%)
Failing to ask about home routines			X		X	X							X		X				5 (27.8%)
Taking the patient into an enclosed room						X	X		X					X	X				5 (27.8%)
Failing to recognize or consider the signs of past trauma/abuse						X	X		X	X				X					5 (27.8%)
Moving too quickly			X			X		X											3 (16.7%)
Failing to recognize the urgency of the patient’s need for care					X			X									X		3 (16.7%)
Trying to do all care at once	X	X																	2 (11.1%)
Failing to ask permission to unbutton his pants			X	X															2 (11.1%)
Labeling the patient as aggressive					X			X											2 (11.1%)
Having too many people in the room during care					X			X											2 (11.1%)
Missing the patient’s non-verbal cues								X	X										2 (11.1%)
Considering chemical restraints too quickly				X	X														2 (11.1%)
Not following or forming a care plan																X	X		2 (11.1%)
Not considering chemical restraints earlier										X									1 (5.6%)
Reapproaching too many times										X									1 (5.6%)
Not reapproaching enough																	X		1 (5.6%)

An X in a cell indicates that the participant reported using the approach listed in the column header.

**Table 3:** Preferred approaches to care (N = 18).

Participant N = 18																			
Approach	Angela	Nicole	Astrid	Alexa	Margaret	Anna	Joy	Sophie	Ella	Bethany	Nancy	Stella	Jesus	Shaina	Donald	Karen	Beatrice	Evangeline	Total (n/%)
PRN medication or scheduling care for after medications take effect				X	X			X	X	X	X	X	X	X			X	X	11 (61.1%)
Wait until patient is comfortable with staff	X	X	X		X	X		X			X	X				X	X	X	10 (55.6%)
Ask for help (different gender)	X	X		X	X	X							X		X	X		X	9 (50%)
Ask for help (colleague with better rapport)	X	X			X	X			X			X	X				X		8 (44.4%)
Ask for help (family)	X	X			X	X					X	X				X		X	8 (44.4%)
Describe procedure and/or risks to declining care		X	X			X	X				X				X	X		X	8 (44.4%)
Non-aggressive touch				X	X		X		X	X						X		X	7 (38.9%)
Bring a support person to help you	X	X			X			X	X				X						6 (33.3%)
Giving options (location/mode of bathing)		X			X			X			X					X			5 (27.8%)
Set up the tools and let patient wash themselves			X					X			X					X		X	5 (27.8%)
Attend to setting events (e.g., hunger)				X			X						X				X	X	5 (27.8%)
Ask for help (finding “the one” staff who can connect with the patient)	X						X	X									X		4 (22.2%)
Divide the bath up into parts	X										X					X	X		4 (22.2%)
Distraction/rewards										X				X			X		3 (16.7%)
Ask for help (doctor)		X				X								X					3 (16.7%)
Interdisciplinary collaboration				X														X	2 (11.1%)
Being ready when patient enters bathroom on their own								X											1 (5.6%)
“Bathing Without a Battle” approaches							X												1 (5.6%)
Do care in the bed								X											1 (5.6%)
Describe your position as senior staff																X			1 (5.6%)

An X in a cell indicates that the participant reported using the approach listed in the column header.

**Table 4:** Risks of incontinence (n = 13).

Participants (n = 13)														
Risks	Angela	Nicole	Astrid	Alexa	Margaret	Anna	Sophie	Ella	Bethany	Stella	Jesus	Donald	Evangeline	Total (n=/%)
Skin breakdown	X	X	X	X	X	X	X	X	X	X	X	X	X	13 (100%)
Infection		X			X	X	X	X	X	X	X	X	X	10 (76.9%)
Impacting the patient’s health (general)	X		X		X		X						X	5 (38.5%)
Sepsis						X					X	X		3 (23.1%)
Social impacts					X	X							X	3 (23.1%)
Prolonged hospital stay	X										X			2 (15.4%)
Death											X	X		2 (15.4%)
Mood								X					X	2 (15.4%)
Prolonging treatment	X													1 (7.7%)

**Table 5:** Rationales for working fast (n = 8).

Participants (n = 8)									
Rationale	Angela	Nicole	Astrid	Alexa	Margaret	Sophie	Ella	Jesus	Total (n=/%)
To get it over with	X		X		X	X		X	5 (62.5%)
Tasks on acute care floors must be done quickly		X		X				X	3 (37.5%)
Less traumatizing for the patient			X			X		X	3 (37.5%)
Less traumatizing to the staff			X						1 (12.5%)
To reassure the patient				X					1 (12.5%)
To be efficient	X								1 (12.5%)
Unsure							X		1 (12.5%)

## Data Availability

The raw data supporting the conclusions of this article will be made available by the authors upon request.

## Supplementary Materials

The following supporting information can be downloaded at: https://www.mdpi.com/article/10.3390/healthcare13131562/s1, Table S1: Consolidated criteria for reporting qualitative studies (COREQ): 32-item checklist; Table S2: Video script and changes based on feedback; Table S3: Interview guide.
