# Supplementary material for: “Heartbreaking, Hardest Part of the Job”: A Qualitative Descriptive Study of Acute Care Nurses’ Work with Patients with Dementia Who Self-Neglect Their Hygiene
Source: Healthcare (Basel). 2025 Jun 30;13(13):1562. doi: 10.3390/healthcare13131562 (PMC12250512; doi:10.3390/healthcare13131562)
Supplement: Supplementary file 1 [file healthcare-13-01562-s001.zip › healthcare-3670428-supplementary.pdf]

Supplementary Materials

Table S1: Consolidated criteria for reporting qualitative studies (COREQ): 32-item checklist

**No. Item**	**Guide questions/description**	**Reported on Page #**
**Domain 1: Research team and reflexivity**		
Personal Characteristics		
1. Interviewer/facilitator	Which author/s conducted the inter view or focus group?	"The interviews were conducted by the lead author, who identifies as a woman. She is a Registered Nurse (RN) and a PhD-prepared faculty member in a Canadian School of Nursing."
2. Credentials	What were the researcher's credentials? E.g. PhD, MD	As above
3. Occupation	What was their occupation at the time of the study?	As above
4. Gender	Was the researcher male or female?	As above
5. Experience and training	What experience or training did the researcher have?	"She has researched and practiced in the area of gerontological nursing for over ten years."
Relationship with participants		
6. Relationship established	Was a relationship established prior to study commencement?	"The researchers had no prior therapeutic or supervisory relationship with any participant."
7. Participant knowledge of the interviewer	What did the participants know about the researcher? e.g. personal goals, reasons for doing the research	"Participants were informed that the research team was conducting interviews about the clinical reasoning involved in working with patients with dementia in hospital settings who decline assistance with hygiene care."
8. Interviewer characteristics	What characteristics were reported about the inter viewer/facilitator? e.g. Bias, assumptions, reasons and interests in the research topic	"As an RN, the lead author shared a professional identity with the participants that may have facilitated rapport and open dialogue." … "While her clinical and academic expertise in dementia care provided valuable contextual understanding, it also carried the risk of influencing how

		participants' responses were interpreted."

Domain 2: study design		
Theoretical framework		
9. Methodological orientation and Theory	What methodological orientation was stated to underpin the study? e.g. grounded theory, discourse analysis, ethnography, phenomenology, content analysis	"This study was a qualitative descriptive design. In this study, Think-Aloud interviewing was employed as a data collection method to explore nurses' decision-making processes in the context of providing hygiene care to patients with dementia who self-neglect and decline assistance."
Participant selection		
10. Sampling	How were participants selected? e.g. purposive, convenience, consecutive, snowball	"Participants were recruited using purposive sampling to ensure a range of experiences among Registered Nurses (RNs) working in acute care hospitals."
11. Method of approach	How were participants approached? e.g. face-to-face, telephone, mail, email	"Recruitment was done through a promotional flyer posted on popular social media sites (Facebook and Instagram), as well as snowball sampling technique"
12. Sample size	How many participants were in the study?	"Thirteen RNs and five Licensed/Registered Practical Nurses (LPN/RPNs) volunteered to participate in this study."
13. Non-participation	How many people refused to participate or dropped out? Reasons?	"There was no attrition and no one declined to particiate after the informed consente process was carried out"
Setting		
14. Setting of data collection	Where was the data collected? e.g. home, clinic, workplace	"Participants joined from private locations of their choosing, ensuring confidentiality and comfort during

		interviews. The interviewer conducted interviews from her office on campus with a blurred virtual background.”
15. Presence of non-participants	Was anyone else present besides the participants and researchers?	“No one else was present during the interviews.”
16. Description of sample	What are the important characteristics of the sample? e.g. demographic data, date	See Table 1
Data collection		
17. Interview guide	Were questions, prompts, guides provided by the authors? Was it pilot tested?	Supplementary material 2
18. Repeat interviews	Were repeat inter views carried out? If yes, how many?	“Credibility was enhanced through member checking, where participants were invited to review summaries of their interview data. While no participants chose to engage in this optional review, the opportunity to participate in this validation process was made available to all. No repeat interviews were performed.”
19. Audio/visual recording	Did the research use audio or visual recording to collect the data?	“Interviews lasted between 45 and 75 minutes, were audio-recorded with consent, and transcribed verbatim.”
20. Field notes	Were field notes made during and/or after the inter view or focus group?	“Field notes were recorded after each interview and memos were written at key stages throughout the analysis process to support transparency in analytic decisions.”
21. Duration	What was the duration of the inter views or focus group?	“Interviews lasted between 45 and 75 minutes”
22. Data saturation	Was data saturation discussed?	“Recruitment and interviewing continued until thematic sufficiency was achieved, which was defined as the point at which no new conceptual insights were emerging from the data”
23. Transcripts returned	Were transcripts returned to participants for comment and/or correction?	“Credibility was enhanced through member

		checking, where participants were invited to review summaries of their interview data. While no participants chose to engage in this optional review, the opportunity to participate in this validation process was made available to all”
**Domain 3: analysis and findings**		
Data analysis		
24. Number of data coders	How many data coders coded the data?	“The first author led the analysis… Codes were then grouped into categories and refined into themes by two independent researchers …”
25. Description of the coding tree	Did authors provide a description of the coding tree?	“Codes were then grouped into categories and refined into themes by two independent researchers (the first and second authors) and represented hierarchically using coding trees. Trees were combined through discussion and consensus with the entire research team.”
26. Derivation of themes	Were themes identified in advance or derived from the data?	“The first author led the analysis, which involved multiple readings of the transcripts, development of initial codes, and constant comparison across interviews”
27. Software	What software, if applicable, was used to manage the data?	“MAXQDA (Verbatim Inc.) was used to assist with data management and coding.”
28. Participant checking	Did participants provide feedback on the findings?	**“**While no participants chose to engage in this optional review, the opportunity to participate in this validation process was made available to all.”
Reporting		
29. Quotations presented	Were participant quotations presented to illustrate the themes/findings? Was each quotation	e.g. “Nicole described the patient as ‘having a shift,

	identified? e.g. participant number	just a natural shift with the sundowning'"
30. Data and findings consistent	Was there consistency between the data presented and the findings?	**e.g. Theme One (Non-preferred approaches to care); approaches are described in narrative form and represented in Table Two**
31. Clarity of major themes	Were major themes clearly presented in the findings?	**Themes one-six**
32. Clarity of minor themes	Is there a description of diverse cases or discussion of minor themes?	**e.g. fecal incontinence and safety concerns as a minor theme; descriptions of multiple participants' perspectives on how long it is appropriate to wait to change incontinence products**

Table S2: Video script and changes based on feedback

	Video Guide
Scene details	**Cast:**<br>• Two RNS, dressed in white and black<br>• One LPN, dressed in royal blue<br>• One PCA, dressed in powder blue<br>**Scene details:** Nursing station on an acute care unit in a hospital. Lights are dimmed and the area is quiet. Paper chart binders are visible in the corner of the room and there are several computers. There is a tape recorder sitting on the desk by one of the computers. There are antiseptic wipes, pens, medical tape, bits of paper, alcohol swabs, etc. piled about (tidy, but cluttered). There are signs posted ("RN vacation requests to be submitted to unit manager by April 30th"; "please review new visitation guidelines for patients on precautions for Covid") and a continuing education article or two tacked up, as well as an obit printed off and tacked up. Three staff members are sitting in mismatched office chairs (LPN, PCA, RN1). PCA is wiping off keyboards etc. with antiseptic wipes. Their bags are at their feet and their coat is slung over the back of the chair. RN1 is sipping a coffee and looking tired. The LPN is sitting slightly in their chair, checking their watch intermittently. They are chatting and waiting for the day staff. They all look rumpled.<br>**Discourses to highlight:**<br>• Cleanliness—commonsense reference to not being able to leave a patient soiled; mention body odour or objective signs of dishevelment<br>• Resistance—set the sim on the night shift, 'battle'/'survival'/'war' metaphors are really common in descriptions of the 'graveyard shift'<br>• Kind coercion—focus on coercive measures as the 'kindest' option<br>• Staff safety—include reference to previous workplace injuries as a backdrop for interaction<br>• Personhood and dementia-- highlight the role of rationality/inability to reason with the patient as a key context for the pursuit of kind coercion
**Dialogue**	**LPN**: I don't know how you're drinking coffee right now. I'd never get to sleep<br>**RN1:** I still have to get the kids off to school when I get home. I won't get to bed until at least 10. By the time I'm done cleaning up the kitchen and getting everyone

out the door, anyway. And I was thinking of going to the 9 am spin class at that new studio

PCA: That sounds awful. I can't wait to pop a sleeping pill and fall into my bed. I went to the gym once after a night shift and I fell asleep on the elliptical

LPN: [Laughs]. Yeah, no. I can't wait to get to bed once I get the kids off.

Call bell rings; LPN answers the phone

LPN: "Hello, can I help you"? [listens] "Ok, I'll check when you're due and bring it down if I can." [Hangs up phone.] To other staff: "Beatrice in room 6 wants something for back pain. Poor woman—she was awake on every round last night. I'm gonna go check and see what she can have and bring it down" [departs room].

[**RN2** walks onto the unit looking fresh, with a coffee in their hands, and stows their purse/bag under the cabinet in the corner. Picks up a piece of paper and pen to make notes and looks at the assignment book]

RN2 to the room: How was the night?

RN1: Oh, busy. You know. Not too bad I guess considering what it's been like in here. We did have one incident with the gentleman in 9B [eyes PCA with a meaningful look].

RN2: Oh yeah? What was his deal? He's got dementia, right?

PCA: Yeah, supposedly not that advanced but I don't know… His brother is looking after him at home and it didn't seem like a great situation.

RN2: Oh yeah? It seemed like he had a pretty good day yesterday. I kind of wondered why he was even here. Seemed a bit mixed up but really nice and he's up and around pretty good.

RN1: Yeah, nice until the sun goes down and then you have to change your approach entirely. Pffft. I went in to do my checks and he let me change his dressing on that big skin tear on his right shoulder; he was all nice and easy to get along with, and then I said we should get to the washroom and get him washed up for bed and he was Dr. Jekyll/ Mr Hyde all of a sudden.

RN2: Oh yeah? What happened?

RN1: You'll hear about it on taped report, but basically I did the dressing on his shoulder and took his vitals and did my checks and everything was fine. Then I got some towels and stuff together and said "let's go get cleaned up for bed" and he starts going into the bathroom and I go to follow him in and he loses it. Starts screaming "Get away from me! Get away from me! Get outta here now. Don't touch me!" and going on and on, you know? Then **PCA** hears and comes in and says, you know, "we're just gonna help you get washed and changed for bed" and tries to take his arm to lead him in

PCA: And it's not like I was being rough, and I had already been in to check on him earlier and we had been joking around and we got on really well then. And then he just whips around and takes a swing at me

RN2: What? Oh no!

RN1: No warning. Nothing.

PCA: I mean, he said no and everything but the intensity came out of nowhere, like he was perfectly nice and then said 'no' super gently and then BAM he was aggressive.

RN1: And so we backed right off and left him, right? And then I went back twice and asked him if he wanted to get ready for bed and he was really nice both times. First time he just said no, so I left it again and then the second time he was like "oh sure, dear, whatever you think" and he even followed me into the bathroom and washed his face fine. Then I asked him to change his pants and he sort of stared at me, so I went to help him with the button and he started swinging. Tried to bite me. It was awful. And I'm thinking "are you kidding me? You literally just said 'Oh sure' and called me dear, and now you're trying to bite me?" No thanks. And I'm trying to be like "Mr. Palmer, I'm just trying to help" and there was no reasoning with him. I'm not gonna be dealing with another workplace injury because he doesn't want to change his underwear.

	**RN2:** Right. We don't want anyone getting hurt. I didn't have him on my assignment yesterday, just saw him when Cheryl needed help turning his roommate. He seemed nice enough then. I wonder how they got him washed yesterday?<br>**PCA:** I guess those are the same clothes he's been wearing since they admitted him the night before last. I think he's still in the same pull-up he was wearing when he was admitted. We don't even have pull-ups here and his brother didn't bring in any extra. I checked his locker and through all of his stuff, and no pull-ups to be found. And he is definitely wearing a pull-up.<br>**RN2:** Oh no. I wonder why they didn't get him washed up yesterday?<br>**PCA:** Who knows? They got busy and they worked so short yesterday. Maybe he refused then too. But he's really starting to smell bad, like with BO<br>**RN1:** Yeah, really bad. It is not a pleasant odeure in there. So I don't know if he will be better for the day shift or not, but if not, you may have to call for something to calm him down before you get in there. Dr. Angelou's on call; he's usually pretty good.<br>**PCA:** I just saw Amir go in to the locker room so he'll be out in a minute. [to RNs] Mind if I skedaddle?<br>**RN1:** No, no, go ahead. [to RN2] It's all on report, anyway, and otherwise it was a pretty uneventful night. LPN is just down with Beatrice Potter in room 6 getting her something for pain, and we had to give Matthew in 4A two units of PRBCs because his HGB was 52 last evening. He had IV Lasix post. Dr. McKinnon was already in and assessed him this morning and he's breathing better. Oh, and we had to change the dressing in 8C at 0300 because it was saturated, but it's only ordered OD and it was already done yesterday morning. I didn't call anyone about it because it's draining sero-sang, and it looks healthy. You'll have to revisit whether OD is enough today though.<br>RN2: Sounds good. You should go. Get some sleep!<br>RN: Thanks. [looking tired]. [Wryly] Have a good day. At least you're not working short!<br>Closing scene details: RN1 makes to depart. RN2 (who was making notes during RN1's discussion) puts her assignment sheet in her pocket, crosses the nursing station and turns on the lights. She takes a seat in front of the computer, logins in, and starts to gather her hair into a ponytail.

Changes to the Script Based on Feedback

Twelve reviewers from different sectors reviewed the video script for conceptual fidelity. Three were simulation experts, four were acute care nurses working in hospitals at the time the script was written, three were nurses who worked in acute care and long-term care settings at the time of review, and two were experts in gerontology. The reviewers held a mix of professional designations to ensure that the simulation adequately captured the scope of practice of Registered Nurses, Licensed Practical Nurses, and Personal Support Workers. In response to reviewers' feedback, we made the following changes to the original script:

1. The focus of the simulation was, initially, a scenario in which the patient had been incontinent of stool and needed assistance with washing up. A reviewer with a gerontological nursing background suggested that we shift the focus toward more routine, everyday care. While incontinence care is a common site for forced care in the context of dementia, the reviewer cautioned that participants may move toward forced care as the only reasonable option too readily when primed with this scenario. The opportunity for critical dialogue might be foreclosed too easily in the case of stool incontinence, because of the health threats to surrounding patients. We shifted the focus of the script toward everyday washing up and dressing instead.
2. Initially, the script included an exchange between the two RNs, with the outgoing RN encouraging the oncoming RN to "put your safety first" when getting his washed today. This line was removed because it was felt that this line might prime participants to default to this as the "correct" way to behave in this scenario, again foreclosing the opportunity for critical dialogue.

3. We included an explicit mention of the patient's dementia diagnosis, on the advice of a simulation expert, so that it would be clear to participants that this simulation focused on dementia care.
4. We included a point of contention in the retelling of the events, where the PSW dissents slightly from the details shared by the RN and provides additional contextualizing details to the story. The RN notes that Mr. Palmer gave them "no warning. Nothing" and the PSW follows up by saying "I mean, he said no and everything but the intensity came out of nowhere." The value in adding this point of contention was in recognizing the role of the PSW as an expert in their work and in building up the co-constructive and collaborative sense-making piece of the script

Table S3: Interview Guide

1. What is your interpretation of what is going on with the patient who declined assistance with care?
2. What are your goals of care in working with this patient?
3. How would you approach this patient to accomplish those goals?
4. What kind of additional information, if any, would you seek out from this patient or about this patient?
5. What do you think would happen if you didn't get this patient washed up on your shift?
6. If the patient continually said "no" or otherwise declined your assistance with care, what would you do?
7. Are there any instances in which your approach to the patient would change? If so, how?
